# Supplementary material for: Immobilization of Alcalase on Silica Supports Modified with Carbosilane and PAMAM Dendrimers
Source: Int J Mol Sci. 2022 Dec 17;23(24):16102. doi: 10.3390/ijms232416102 (PMC9783553; doi:10.3390/ijms232416102)
Supplement: Supplementary file 1 [file ijms-23-16102-s001.zip › ijms-2082701-supplementary.pdf]

SUPPLEMENTARY MATERIAL

Immobilization of Alcalase on silica supports modified with carbosilane and PAMAM dendrimers

María Sánchez-Milla [1,2,3,†], Ester Hernández-Corroto [4,†], Javier Sánchez-Nieves [1,2,3,5], Rafael Gómez [1,2,3,5], María Luisa Marina [3,4], María Concepción García [3,4,*] and F. Javier de la Mata [1,2,3,5,*]

1 Universidad de Alcalá, Departamento de Química Orgánica y Química Inorgánica, Ctra. Madrid-Barcelona Km. 33.600, 28871 Alcalá de Henares, Madrid, Spain.

2 Networking Research Center on Bioengineering, Biomaterials and Nanomedicine (CIBER-BBN), Madrid, Spain

3 Universidad de Alcalá, Instituto de Investigación Química "Andrés M. del Río", Ctra. Madrid-Barcelona Km. 33.600, 28871 Alcalá de Henares, Madrid, Spain

4 Universidad de Alcalá, Departamento de Química Analítica, Química Física e Ingeniería Química, Ctra. Madrid-Barcelona Km. 33.600, 28871 Alcalá de Henares, Madrid, Spain.

5 Instituto Ramón y Cajal de Investigación Sanitaria (IRYCIS).

* Correspondence: concepcion.garcia@uah.es (M.C.G.); javier.delamata@uah.es (F.J.d.l.M.)

† These authors contribute equally to this work.

Table S1. Codes and names of different supports used for the immobilization of Alcalase.

Code	Support name	Type of support
**1-SiO_2**	SiO_2-L-NH_2	Linear molecule
**2-SiO_2**	SiO_2-G_0Si-NH_2	Carbosilane dendrimer
**3-SiO_2**	SiO_2-G_1Si-NH_2	Carbosilane dendrimer
**4-SiO_2**	SiO_2-G_1O_3-NH_2	Carbosilane dendrimer
**5-SiO_2**	SiO_2-G_0PAMAM-NH_2	PAMAM dendrimer
**6-SiO_2**	SiO_2-G_1PAMAM-NH_2	PAMAM dendrimer
